# Catalase Influence in the Regulation of Coronary Resistance by Estrogen: Joint Action of Nitric Oxide and Hydrogen Peroxide

**DOI:** 10.1155/2014/159852

**Published:** 2014-02-06

**Authors:** Paulo C. Schenkel, Rafael O. Fernandes, Vinícius U. Viegas, Cristina Campos, Tânia R. G. Fernandes, Alex Sander da Rosa Araujo, Adriane Belló-Klein

**Affiliations:** Laboratório de Fisiologia Cardiovascular, Departamento de Fisiologia, Instituto de Ciências Básicas da Saúde, Universidade Federal do Rio Grande do Sul, Sarmento Leite 500, Bairro Farroupilha, 90050-170 Porto Alegre, RS, Brazil

## Abstract

We tested the influence of estrogen on coronary resistance regulation by modulating nitric oxide (NO) and hydrogen peroxide (H_2_O_2_) levels in female rats. For this, estrogen levels were manipulated and the hearts were immediately excised and perfused at a constant flow using a Langendorff's apparatus. Higher estrogen levels were associated with a lower coronary resistance, increased nitric oxide bioavailability, and higher levels of H_2_O_2_. When oxide nitric synthase blockade by L-NAME was performed, no significant changes were found in coronary resistance of ovariectomized rats. Additionally, we found an inverse association between NO levels and catalase activity. Taken together, our data suggest that, in the absence of estrogen influence and, therefore, reduced NO bioavailability, coronary resistance regulation seems to be more dependent on the H_2_O_2_ that is maintained at low levels by increased catalase activity.

## 1. Introduction

Decreased levels of estrogen play a critical role in heart disease development after menopause. The loss of ovarian hormones has a widespread adverse impact, increasing the risk of cardiovascular events, such as myocardial infarction (MI) [1–3].

The ischemic insult caused by MI can be increased during the early moments of reperfusion, referred to as ischemia/reperfusion (I/R) injury [4]. Moreover, myocardial cells tissue damage is aggravated by producing reactive oxygen species (ROS) under ischemic conditions by mitochondrial electron transport chain or by enzymes such as NADPH oxidase, xanthine oxidase, and nitric oxide synthase (NOS) [5].

Under I/R situations, such as in acute MI, endothelial injury decreases the production of nitric oxide (NO) [6], and NOS can become a source of superoxide anion [7, 8]. This process is termed NOS uncoupling. Moreover, NO produced from NOS can react with superoxide anion decreasing NO bioavailability and generating the potent oxidant, peroxynitrite (ONOO−) [9]. On the other hand, Yada et al. demonstrated that endogenous hydrogen peroxide (H_2_O_2_) contributes to coronary vasodilatation during I/R *in vivo* as a compensatory mechanism for the NO loss [10]. And, according to Cosentino et al. [11], H_2_O_2_ can be involved in vasorelaxation during uncoupling of NO synthesis. Moreover, it has been shown that H_2_O_2_ has a critical role as a signaling molecule for cardiac remodeling, probably due to its greater stability and permeability as compared to other ROS. In this context, the H_2_O_2_ levels modulation by antioxidant enzyme catalase (CAT) seems to play an important role in cardiovascular function [11].

Estrogen therapy (ET) has been considered as a means to reduce cardiovascular risk in postmenopausal women [1] and in animals submitted to I/R injury [12–14]. Furthermore, it has been reported that ET improves NO-mediated vasodilatation in ovariectomized rats [15]. However, the ET effects on H_2_O_2_ concentrations and its repercussion in coronary tone are not known.

Therefore, the aim in this study was to verify if the influence of estrogen over coronary resistance is modulated by hydrogen peroxide concentrations through catalase activity control. Another aim of this study was to determine, through the NOS blockade, whether NO participates of this mechanism.

## 2. Methods

### 2.1. Animals

Twenty-nine female Wistar rats (60 days, 200 ± 20 g) were obtained from the Central Animal House at Universidade Federal do Rio Grande do Sul, Brazil. The animals were housed in plastic cages (four animals each) and received water and food *ad libitum*. They were maintained under standard laboratory conditions (controlled temperature of 21°C, 12 hours light/dark cycle). They were divided into three groups: SHAM, that was submitted to a sham surgery of bilateral ovariectomy; OVX, which was ovariectomized; and OVX + E_2_, that was ovariectomized and received 17*β*-estradiol replacement. Each group was divided into two groups for the isolated heart perfusion, according to the perfusion solution utilized (Tyrode or Tyrode + L-NAME). Thus, the six experimental groups were TYRODE-SHAM (*n* = 5); L-NAME-SHAM (*n* = 4); TYRODE-OVX (*n* = 6); L-NAME-OVX (*n* = 4); TYRODE-OVX + E_2_ (*n* = 4); L-NAME-OVX + E_2_ (*n* = 4).

### 2.2. Ethical Approval

The experimental design was approved by the Committee on Animal Care and Use of the Universidade Federal do Rio Grande do Sul, following the Principles of Laboratory Animal Care published by the Council for International Organizations of Medical Science.

### 2.3. Ovariectomy (OVX)

The rats were anesthetized (ketamine 90 mg kg^−1^; xylazine 10 mg kg^−1^ i.p.) and bilateral ovariectomy or sham operation was performed. After a week, the animals were submitted to a 17*β*-estradiol replacement or replacement simulation.

### 2.4. 17*β*-Estradiol Therapy

Briefly, 15 mm medical grade tubing (1.02 mm i.d. × 2.16 mm o.d.) was filled with 10 *μ*L of 5% (w : v) 17*β*-estradiol (Sigma Chemical Co., St. Louis, MO, USA) in sunflower oil and sealed with silicone. Capsules were soaked in sterile saline overnight and implanted subcutaneously between the scapulae under anesthesia. Sham animals were implanted with capsules containing just sunflower oil [16].

### 2.5. 17*β*-Estradiol Concentration

Blood samples were collected 28 days after ovariectomy surgery by the retrorbital venous plexus and immediately centrifuged at 1000 g for 10 min. The plasma 17*β*-estradiol concentration was estimated by chemiluminescence using the Immunolite 2000 apparatus (Biomedical Technologies Inc. Strougerton, MA, USA) at Weinmann Clinical Analysis Laboratory. The results were expressed as pg mL plasma^−1^.

### 2.6. Estrous Cycle Determination

In female rats not submitted to ovariectomy, cycle determination was started at the 28th day of the experimental protocol. The cycle of each female rat was determined by observation of vaginal smears, which were taken using plastic tip. Saline was placed on the vaginal opening, aspirated, and then placed on a microscopic slide. Animals in the diestrus phase were used [17].

### 2.7. Experimental Protocol

After 28 days of the ovariectomy, the rats were killed by decapitation and the hearts were immediately excised and perfused at a constant flow using a Langendorff's apparatus [18]. After the connection of aorta and insertion of the balloon into left ventricle, the hearts were stabilized for 20 min with an end-diastolic pressure set to about 10 mmHg. Animals that did not show stable conditions at the end of this period were discarded. Global ischemia was induced by suspending the coronary flow for 30 min, and after that hearts were reperfused for 20 min.

### 2.8. Isolated Heart Perfusion

The hearts were rapidly excised through a median sternotomy and aorta was retrogradely perfused (Langendorff model) using a Langendorff apparatus (Hugo Sachs Electronics, March-Hugstetten, Germany). The isolated hearts were perfused with two modified Tyrode solutions. One solution containing 120 mmol L^−1^ NaCl, 5.4 mmol L^−1^ KCl, 1.8 mmol L^−1^ MgCl_2_, 1.25 mmol L^−1^ CaCl_2_·2H_2_O, 2 mmol L^−1^ NaH_2_PO_4_, 27 mmol L^−1^ NaHCO_3_, 1.8 mmol L^−1^ Na_2_SO_4_, and 11 mmol L^−1^ glucose, and, other containing similar composition plus 100 *μ*mol L^−1^ N^*ω*^-nitro-L-arginine methyl ester (L-NAME) in a doses capable to inhibit all NOS isoforms [19]. Both solutions were equilibrated with a 95% oxygen and 5% carbon dioxide mixture to give a pH of 7.4 and perfused at a rate of 10 mL min^−1^ with a peristaltic pump (MS-Reglo 4 channels, Hugo Sachs Electronics) and kept at 37°C. A latex balloon was introduced into the left ventricle via the left atrium and was pressurized with a spindle syringe until it reached a preload of 10 mmHg to standardize cardiac work load. Heart rate (HR) and left ventricular end diastolic pressure (LVEDP) as well as the left ventricular developed pressure (LVDP, systolic minus end diastolic pressure) and coronary perfusion pressure (CPP) were monitored with a TPS Statham transducer and used to assess cardiac function.

### 2.9. Tissue Preparation

After perfusion protocol, the hearts were weighed and homogenized (1.15% w/v KCl and phenyl methyl sulphonyl fluoride PMSF 20 mmol L^−1^) in Ultra-Turrax. The suspension was centrifuged at 600 g for 10 min at 0–4°C to remove the nuclei and cell debris [20] and supernatants were used for the assay of nitric oxide metabolism and enzymatic activity. Cardiac tissue samples were rapidly removed after perfusion protocol and frozen at −80°C for the evaluation of hydrogen peroxide steady state concentration.

### 2.10. Determination of Nitrates (NO_3_^−^) and Nitrites (NO_2_^−^)

Nitrites were determined using the Griess reagent, in which a chromophore with a strong absorbance at 540 nm is formed by reaction of nitrite with a mixture of naphthyl-etilenediamine (0.1%) and sulphanilamide (1%). The absorbance was measured in a spectrophotometer to give the nitrite concentration. Nitrates were determined as total nitrites (initial nitrite plus nitrite reduced from nitrate) after its reduction using nitrate reductase, from *Aspergillus* species in the presence of NADPH. A standard curve was established with a set of serial dilutions (10^−8^–10^−3^ mol L^−1^) of sodium nitrite. Results were expressed as mmol L^−1^ [21].

### 2.11. Determination of Hydrogen Peroxide

The assay was based in horseradish peroxidase (HRPO)-mediated oxidation of phenol red by hydrogen peroxide, leading to the formation of a compound that absorbs at 610 nm. Tissues were incubated for 30 min at 37°C in phosphate buffer 10 mmol L^−1^, NaCl 140 mmol L^−1^, and dextrose 5 mmol L^−1^. The supernatants were transferred for tubes with phenol 0.28 mmol L^−1^ and 8.5 U mL^−1^ HRPO buffer, where, after 5 min incubation, NaOH 1 N was added and the mixture was read at 610 nm. The results were expressed as nmoles g tissue^−1^ [22].

### 2.12. Determination of Antioxidant Enzyme Activities

Superoxide dismutase (SOD) activity, expressed as U mg protein^−1^ of protein, was based on the inhibition of superoxide radical reaction with pyrogallol [23]. Catalase (CAT) activity was determined by following the decrease in 240 nm absorption of hydrogen peroxide (H_2_O_2_). It was expressed as nmoles mg protein^−1^ [24].

### 2.13. Determination of Protein Concentration

Protein was measured by the method of Lowry et al. [25], using bovine serum albumin as standard.

### 2.14. Statistical Analysis

Data were expressed as mean ± S.D. and compared using two way ANOVA followed by Student-Newman-Keuls multiple comparison test. Values of *P* < 0.05 were considered significant.

## 3. Results

### 3.1. 17*β*-Estradiol Concentration and Body Weight

17*β*-estradiol level was reduced significantly in OVX group (14.3 ± 2.1 pg mL^−1^) as compared with SHAM, in diestrus phase (30.7 ± 5.6 pg mL^−1^), and higher in OVX + E_2_ group (63.3 ± 2.8 pg mL^−1^). Body weight showed an opposite profile to 17*β*-estradiol concentration. OVX rats had significantly higher body weight than SHAM and OVX + E_2_ (252 ± 10; 232 ± 9; and 210 ± 7 g, resp.).

### 3.2. Isolated Heart Perfusion

**Figure 1:**
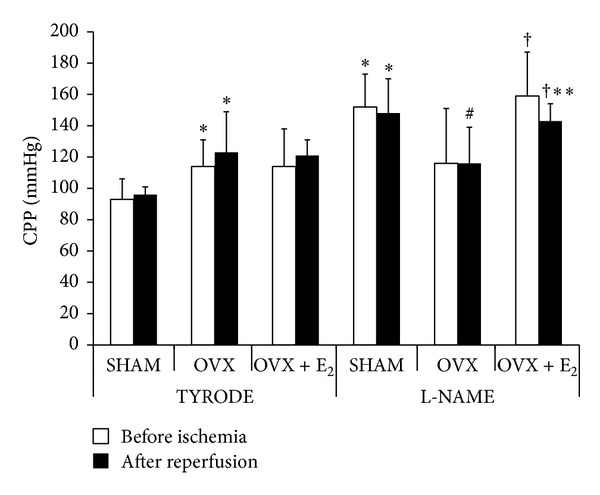
Coronary perfusion pressure (CPP) (in mmHg) of the different experimental groups before ischemia and after reperfusion. Values are expressed as mean ± S.D. of 4–6 animals/group. *Significantly different from TYRODE-SHAM (*P* < 0.05); ^#^significantly different from L-NAME-SHAM (*P* < 0.05); ^†^significantly different from TYRODE-OVX + E_2_ (*P* < 0.05); **significantly different from L-NAME-OVX (*P* < 0.05).

Tyrode-OVX rats have shown a significant increase in CPP (32% and 28%, resp.) before ischemia and after reperfusion when compared to SHAM. CPP did not differ significantly in OVX + E_2_ group before ischemia as compared to SHAM and OVX groups perfused with Tyrode (Figure 1). When NOS blockade by L-NAME was performed, CPP was increased in SHAM (54% and 55%) and in OVX + E_2_ (22% and 18%) groups, respectively, before ischemia and after reperfusion as compared to their respective Tyrode control groups (Figure 1). The OVX groups showed no significant differences in CPP when comparing Tyrode and L-NAME perfusion. No significant differences were found in contractile function parameters (Table 1).

### 3.3. Determination of Nitrates (NO_3_^−^) and Nitrites (NO_2_^−^) and Hydrogen Peroxide (H_**2**_O_**2**_)

**Figure 2:**
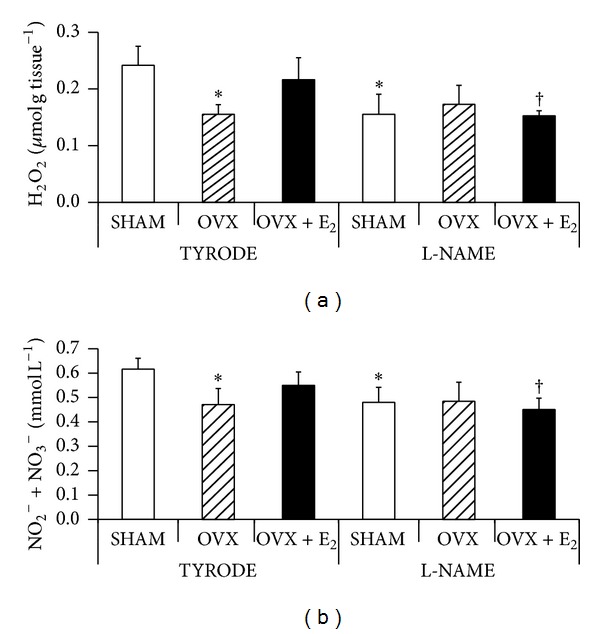
Hydrogen peroxide (H_2_O_2_) concentration (in *μ*mol g tissue^−1^) in cardiac tissue slices (a) and nitrites (NO_2_^−^) and nitrates (NO_3_^−^) concentration (in mmol L^−1^) in cardiac muscle homogenates (b) of the different experimental groups at the end of reperfusion. Values are expressed as mean ± S.D. of 4–6 animals/group. *Significantly different from TYRODE-SHAM (*P* < 0.05); ^†^significantly different from TYRODE-OVX + E_2_ (*P* < 0.05).

Nitrates/nitrites and hydrogen peroxide levels have shown the same profile of oscillation (Figures 2(a) and 2(b)). In animals perfused with Tyrode, both parameters were lower 35% in the OVX when compared to SHAM. In OVX + E_2_ these parameters increased 17% and 34%, respectively, in comparison to OVX (Figures 2(a) and 2(b)). The L-NAME SHAM and OVX + E_2_ groups showed a decrease in the nitrates/nitrites levels by 22% and 18%, respectively, and H_2_O_2_ levels by 36% and 30%, respectively, as compared to their respective Tyrode control groups. No significant differences were found in these parameters evaluated in OVX groups.

### 3.4. Determination of Antioxidant Enzyme Activities

In the Tyrode perfused groups, CAT activity was elevated in the OVX group as compared to SHAM and was restored in OVX + E_2_ group (Table 2). In the L-NAME perfused groups, SHAM and OVX + E_2_ showed an increased CAT activity (19% and 31%, resp.), when compared to their respective Tyrode control groups, reaching similar values than those observed in OVX groups, that did not exhibit alterations according to the solution utilized. No significant differences were found in SOD activity in the different experimental groups.

## 4. Discussion

The main finding of this study was to demonstrate that the protective role of estrogen in attenuating increased coronary resistance seems to be due to NO influence on the modulation of CAT activity and, by this way, regulating H_2_O_2_ concentration.

As expected, low estrogen levels were associated with higher coronary resistance in OVX group. The decrease in estrogen levels seems to impair the vascular tone regulation and, thus, has been associated with higher risk of cardiovascular disease [26]. In fact, an increased incidence of acute MI in postmenopausal women has been observed [27]. This fact, combined with the prior knowledge of H2O2 influence on coronary vascular bed by modulating the activity and expression of eNOS via the PI3-kinase and MAPK in-vitro [28], were the main reason to support our choice to develop this study utilizing the classical ex-vivo I/R model. To analyze the importance of estrogen in the coronary tone regulation in adverse heart situations, we simulated menopause by promoting bilateral ovariectomy and used ET to recover its levels. These procedures were effective in reducing and increasing, respectively, 17*β*-estradiol plasma concentration.

In accordance with the literature, the increase in vascular resistance in ovariectomized rats has been attributed, in part, to a lower NO bioavailability [29]. It has been shown that estrogen may act as an indirect coronary vasodilator mediated by endothelium-derived vasodilatory substances, such as NO [29]. Corroborating these findings, in this study we observed an opposite profile between nitrates/nitrites levels and CPP in the OVX group perfused with Tyrode. It is possible that, with the withdrawal of estrogen influence, endothelial dysfunction takes place, increasing coronary resistance [27]. When NO levels were increased by ET, the coronary resistance was attenuated in Tyrode-OVX + E_2_ group. This important role of estrogen in modulation of vascular tone appears to be mediated by PI3-kinase/Akt pathway in endothelial cells resulting in increased expression of NOS and leading to the greater production of NO [28]. Associated with the decrease in NO bioavailability, the OVX group also showed lower H_2_O_2_ levels as compared to SHAM perfused with Tyrode. The regulation of coronary tone is physiologically modulated in great part by NO. Additionally, numerous other molecules are involved in the regulation of vascular tone. Among these, the more prominent influence of H_2_O_2_ in pathological situations has been highlighted, when NO influence is reduced [11, 30]. Therefore, the control of these substances appears to be an important mechanism involved in coronary tone regulation. Hydrogen peroxide, a downstream ROS also generate by NADPH-oxidase, has been suggested to be a key molecule in the regulation of the coronary tone by stimulating endothelium-dependent and/or endothelium independent vasorelaxation [30]. These effects, mediated by the regulation of smooth muscle's potassium channel and activation of eNOS, respectively, may represent a cellular adaptation in adverse situations, as in I/R [31, 32]. In this context, the estrogen influence in the vascular tone regulation also appears to be determined by less H_2_O_2_ production, since this hormone suppresses the activity and expression of NADPH oxidase [33]. In the present study, Tyrode-OVX group presented reduced H_2_O_2_ levels and higher CAT activity in relation to SHAM group after I/R. Although an inverse association between estrogen and ROS by suppression of the expression and activity of NADPH oxidase has been shown in the literature [33], the lower H_2_O_2_ levels, showed in this study, were associated with increased activity of CAT in ovariectomized group. Our findings corroborate previous findings of Behr et al. that showed an increase in peroxidases CAT and glutathione in ovariectomized rats [34]. A consequent adaptation of antioxidant enzymes appears to modulate the H_2_O_2_ levels and thus contribute to vascular tone regulation in rats with low estrogen levels.

Additionally, our findings suggest that this higher CAT activity seems to be resultant from the lower NO bioavailability in animals with reduced estrogen levels, since this group exhibited lower nitrates/nitrites levels. In fact, the antioxidant activity of CAT seems to be modulated by the NO levels. It has been shown in the literature that NO and CAT can quickly react and form a complex that culminate in reduction of NO bioavailability and reduced activity of catalase [35]. In order to determine the role of NO in the estrogen influence on coronary tone, L-NAME was added to the perfusion liquid. As expected, NOS blockade reduced significantly nitrates/nitrites levels in SHAM and OVX + E_2_ to values similar to those of OVX group. Furthermore, nitrates/nitrites were not modified in OVX groups perfused with Tyrode or L-NAME, reinforcing our suggestion of a reduced influence of NO in the regulation of coronary tone in ovariectomized rats. Associated with this, CAT was more effective to reduce H_2_O_2_ levels in SHAM and OVX + E_2_ groups perfused with L-NAME since its activity became less inhibited by NOS blockade. These results suggest that estrogen exerts its effects by its capacity to influence NOS activity.

Taken together, our data highlight the influence of estrogen on NOS and the CAT contribution in the regulation of NO and H_2_O_2_ levels to an important joint action in the control of coronary resistance. Regulating these parameters by negative feedback mechanism, CAT emerges as a key molecule in the regulatory control of the coronary tone. Moreover, the increase in its activity with reduced NO levels suggests a significant influence of H_2_O_2_ as vasomodulator in adverse situations. This knowledge may be relevant in the proposal of therapeutical strategies capable to diminish the adverse effects observed in the I/R syndrome.

## Figures and Tables

**Table 1:** Contractile function of the different experimental groups before ischemia and after reperfusion.

	Before Ischemia			After Reperfusion		
	HR (bpm)	LVEDP (mmHg)	LVDP (mmHg)	HR (bpm)	LVEDP (mmHg)	LVDP (mmHg)
TYRODE						
SHAM	214 ± 27	10 ± 1	94 ± 21	188 ± 27	58 ± 12	21 ± 9
OVX	201 ± 38	10 ± 1	87 ± 20	176 ± 46	63 ± 23	31 ± 27
OVX + E_2_	184 ± 31	10 ± 1	95 ± 15	169 ± 28	62 ± 24	33 ± 21
L-NAME						
SHAM	202 ± 39	10 ± 1	100 ± 15	190 ± 50	55 ± 21	38 ± 30
OVX	206 ± 36	9 ± 1	84 ± 30	195 ± 31	57 ± 23	32 ± 19
OVX + E_2_	186 ± 42	10 ± 1	99 ± 15	165 ± 58	66 ± 31	36 ± 29

Values are expressed as mean ± S.D. of 4–6 animals/group. Heart rate (HR), left ventricle end diastolic pressure (LVEDP), left ventricular developed pressure (LVDP).

**Table 2:** Antioxidant activity in cardiac muscle homogenates of the different experimental groups at the end of reperfusion.

	SOD (U mg prot.^−1^)	CAT (pmol mg prot.^−1^)
TYRODE		
SHAM	13.3 ± 2.0	22.4 ± 1.7
OVX	11.7 ± 0.9	26.9 ± 0.7*
OVX + E_2_	11.9 ± 1.2	20.8 ± 2.2**
L-NAME		
SHAM	14.4 ± 1.7	26.6 ± 2.0*
OVX	12.0 ± 0.7	27.9 ± 2.6
OVX + E_2_	12.2 ± 2.8	27.4 ± 3.1^†^

Values are expressed as mean ± S.D. of 4–6 animals/group.

*significantly different from TYRODE-SHAM (*P* < 0.05); **significantly different from TYRODE-OVX (*P* < 0.05); ^†^significantly different from TYRODE-OVX + E_2_ (*P* < 0.05).
